# Down Regulation of NO Signaling in *Trypanosoma cruzi* upon Parasite-Extracellular Matrix Interaction: Changes in Protein Modification by Nitrosylation and Nitration

**DOI:** 10.1371/journal.pntd.0003683

**Published:** 2015-04-09

**Authors:** Milton Pereira, Chrislaine Soares, Gisele André Baptista Canuto, Marina Franco Maggi Tavares, Walter Colli, Maria Julia M. Alves

**Affiliations:** 1 Departamento de Bioquímica, Instituto de Química, Universidade de São Paulo, São Paulo, Brazil; 2 Departamento de Química Fundamental, Instituto de Química Universidade de São Paulo, São Paulo, Brazil; McGill University, CANADA

## Abstract

**Background:**

Adhesion of the *Trypanosoma cruzi* trypomastigotes, the causative agent of Chagas' disease in humans, to components of the extracellular matrix (ECM) is an important step in host cell invasion. The signaling events triggered in the parasite upon binding to ECM are less explored and, to our knowledge, there is no data available regarding •NO signaling.

**Methodology/Principal Findings:**

Trypomastigotes were incubated with ECM for different periods of time. Nitrated and S-nitrosylated proteins were analyzed by Western blotting using anti-nitrotyrosine and S-nitrosyl cysteine antibodies. At 2 h incubation time, a decrease in NO synthase activity, •NO, citrulline, arginine and cGMP concentrations, as well as the protein modifications levels have been observed in the parasite. The modified proteins were enriched by immunoprecipitation with anti-nitrotyrosine antibodies (nitrated proteins) or by the biotin switch method (S-nitrosylated proteins) and identified by MS/MS. The presence of both modifications was confirmed in proteins of interest by immunoblotting or immunoprecipitation.

**Conclusions/Significance:**

For the first time it was shown that *T*. *cruzi* proteins are amenable to modifications by S-nitrosylation and nitration. When *T*. *cruzi* trypomastigotes are incubated with the extracellular matrix there is a general down regulation of these reactions, including a decrease in both NOS activity and cGMP concentration. Notwithstanding, some specific proteins, such as enolase or histones had, at least, their nitration levels increased. This suggests that post-translational modifications of *T*. *cruzi* proteins are not only a reflex of NOS activity, implying other mechanisms that circumvent a relatively low synthesis of •NO. In conclusion, the extracellular matrix, a cell surrounding layer of macromolecules that have to be trespassed by the parasite in order to be internalized into host cells, contributes to the modification of •NO signaling in the parasite, probably an essential move for the ensuing invasion step.

## Author Summary

Interaction of *Trypanosoma cruzi* with the extracellular matrix (ECM) is an essential step in the invasion of mammalian cells. However, the nature of the signaling triggered in the parasite is poorly understood. Herein the key role of nitric oxide in *T*. *cruzi* signaling is described, using an ECM preparation, in the absence of host cells. Inhibition of NOS activity, with the expected decrease in •NO production, as well as decrease in cGMP concentration were observed by the incubation of *T*. *cruzi* trypomastigotes with ECM. Additionally, lower levels of protein S-nitrosylation and nitration were detected. These post-translational modifications have been analyzed by biotin-switch and protein immunoprecipitation approaches coupled to mass spectrometry. The presence of both modifications was confirmed for specific proteins, as mucin II (S-nitrosylation), histones, enolase and tubulins. To our knowledge, decrease in the •NO signaling pathway upon *T*. *cruzi* trypomastigotes adhesion to ECM, affecting both the canonical pathway (•NO-soluble guanylyl cyclase-cGMP) and protein S-nitrosylation and nitration is described for the first time in this parasite.

## Introduction

*Trypanosoma cruzi* is the etiological agent of Chagas disease, an infectious disease affecting areas of poor socioeconomic development. The parasite infects a wide range of mammalian hosts, including humans, from which 7–8 million are infected and other 25 million are at risk of contamination [1]. *T*. *cruzi* trypomastigotes, the classical parasite infective form, invade almost all mammalian cells, including macrophages [2,3,4], being exposed to nitrosative and oxidative stress during the life cycle [5,6,7]. The cytotoxic effect of •NO and its derivatives on pathogens such as *T*. *cruzi* is well known.

In mammals and other organisms, the free radical •NO is endogenously synthesized by nitric oxide synthase catalyzing the conversion of L-arginine to L-citrulline [8], a reaction that depends on heme, FAD, FMN and tetrahydro-L-biopterin (BH_4_) as co-factors. •NO is highly reactive towards O_2_, but reactions with biological molecules preferentially occur with •NO- derived species (N_2_O_3,_ NO_2_^•^ or ONOO^-^) [9]. Biologically, •NO plays essential role in cell signaling, acting by two main mechanisms: (i) activation of guanylyl cyclase, yielding cGMP—the classical pathway; or (ii) acting in post-translational modifications such as S-nitrosylation and tyrosine nitration- the non-classical pathway [10,11]. Protein S-nitrosylation and tyrosine nitration affect the activity of many relevant targets of several biological processes [12,13].

Proteins are S-nitrosylated (SNO) by the addition of a nitroso group into a cysteine residue in a non-enzymatic process, dependent on the local nitric oxide concentration or by transnitrosylation, a key mechanism in •NO signaling (acquisition of a •NO from another S-nitrosothiol) [14,15,16]. Denitrosylation may occurs by nonenzymatic mechanisms or by the action of denitrosylases [17,18,19]. New targets of S-nitrosylation are being extensively described in different organisms due to the development of tools such as the traditional biotin-switch technique associated with proteomic analysis [20,21]. As an example, 319 putative S-nitrosylation targets, as well as enzymatic denitrosylating and transnitrosylating activities in *Plasmodium falciparum* were recently described [22]. Of note, *P*. *falciparum* lacks a NOS ortholog and probably produces •NO from a nitrate/nitrite chemical reduction pathway [23].

In contrast to S-nitrosylation, tyrosine nitration of proteins was classically regarded as an undesired byproduct of radical species with greater reactivity capable of oxidizing tyrosine to 3-nitro-tyrosine. However, tyrosine nitration of proteins occurs under physiological conditions, with an increment of 3-nitro-tyrosine in many physiopathological and aging processes. Protein nitration is mediated by free radical reactions, with the intermediate •Tyr reacting with •NO or •NO_2_ [24]. Not all proteins are nitrated, pointing out to the specificity of the modification. Only one or two specific protein residues are preferentially modified and a close relationship between protein tyrosine nitration and the presence of a transition metal has been made [rev. 25]. Although not well established as S-nitrosylation, evidence gathered in the past few years suggests that tyrosine-nitrated proteins regulate several biological processes, such as stress response in plants [26], cytochrome c regulation [27], protein degradation [28], control of the redox environment [29] and PKC signaling [30].

There is a limited knowledge of •NO signaling in *T*. *cruzi*, as happens with other parasites [31]. S-nitrosylation or tyrosine nitration of *T*. *cruzi* proteins remains largely unexplored, despite the relevance of •NO and •NO-derived species produced by mammals in response to *T*. *cruzi* infection. *In vitro* treatment of cruzipain, the major *T*. *cruzi* papain-like cysteine proteinase [32], with •NO donors led to inhibition of the enzyme activity [33], but this modification has not been reported *in vivo*. Additionally, the putative signaling in response to the endogenous •NO formation is mostly unknown in *T*. *cruzi*.

Biochemical evidence of NOS activity, with •NO donors leading to an increase in the cGMP concentration was described in *T*. *cruzi* extracts [34]. Probing with an anti-neuronal NOS antibody the enzyme was localized in the inner surface of cell membranes, cytosol, flagellum and apical extremity [35]. However, NOS and guanylyl cyclase orthologs seem to be lacking in the parasite genome. An adenylyl cyclase containing a putative guanylyl cyclase domain was suggested to be responsible for cGMP production [36,37] and a soluble dual-specificity phosphodiesterase (TcrPDEC), capable of cleaving both cAMP and cGMP with similar Km (20–31.6 μM and 78.2 μM, respectively) would be responsible for the degradation of cGMP [38,39,40,41]. The downstream effector of cGMP is assumed to be the cGMP-dependent protein kinase (PKG). While the involvement of cAMP is relatively well known in biological processes, such as *T*. *cruzi* metacyclogenesis [40,42,43] and downstream proteins that interact with PKA were characterized [44] the knowledge of cGMP signaling is far from being understood. Although the role of cGMP signaling pathway is currently unresolved in *T*. *cruzi* and other kinetoplastids, the presence of cGMP-specific kinase in *T*. *brucei* [45] and *Leishmania* [46] points out to the presence of the pathway in these parasites.

In addition to a structural role in tissues, the extracellular matrix (ECM), a complex tridimensional structure composed of more than 300 proteins and glycoproteins [47], is relevant for many cellular signaling pathways, including •NO signaling in mammalians. *T*. *cruzi* trypomastigotes bind to components of the extracellular matrix (ECM), such as laminin, fibronectin, collagen, heparan sulfate, thrombospondin or galectin-3, as an early event of the infection process of mammalian cells [2,3,48,49]. Despite this, the signaling pathways triggered in *T*. *cruzi* after adhesion to ECM or its components are less characterized. Adhesion to laminin or fibronectin leads to changes in the phosphorylation level of *T*. *cruzi* proteins, including paraflagellar rod proteins and tubulins, probably involving the ERK1/2 pathway [50].

Herein, the role of nitric oxide in post-translational modification of proteins as a consequence of trypomastigotes adhesion to ECM is focused. A decrease of the •NO signaling pathway, including S-nitrosylation and tyrosine nitration of proteins was observed in *T*. *cruzi* trypomastigotes upon adhesion to host cell-derived ECM, an essential event for mammalian host cell invasion. To our knowledge this phenomenon is described for the first time.

## Materials and Methods

### Chemicals

S-methyl-methanethiosulfonate (MMTS), imidazole, L-arginine and L-citrulline, 3-isobutyl-1-methylxanthine (IBMX), GTP, sulfanilamide, N-(1-naphthylethylenediamine dichloride, HgCl_2_, S-nitrosoglutathione, neocupreine and also the antibodies: anti-alfa tubulin, anti-nitrosocysteine, anti-mouse FITC conjugated, anti-rabbit HRP conjugated were purchased from Sigma-Aldrich (St. Louis, USA). Phosphoric acid and sodium hydroxide were acquired from Synth (São Paulo, Brazil). Sodium dihydrogenphosphate was purchased from Merck (Darmstadt, Germany). The antibody anti-nitro-tyrosine was obtained from Millipore (Billerica, USA). Sepharose beads, anti-rabbit Alexafluor 555 conjugated and DAPI were acquired from Invitrogen (Carlsbad, USA). EZ-link HPDP Biotin was from Thermo Scientific (Waltham, USA). The L-[^3^H]-Arginine was from PerkinElmer (Waltham, USA).

### Parasite cultures

*T*. *cruzi* epimastigotes, Y strain, were cultivated at 28°C in Liver infusion Tryptose (LIT) medium supplemented with 10% fetal bovine serum (FBS), up to 10^7^ epimastigotes per mL. [51]. *T*. *cruzi* trypomastigotes, Y strain, were maintained by infection in LLC-MK_2_ cells in DMEM supplemented with 2% FBS at 37°C and 5% CO_2_. Five days after infection, trypomastigotes released into the medium were collected, washed in DMEM 2% FBS (10,000 x G for 12 minutes) and resuspended to adequate cell density accordingly to the experiment [52].

### Parasite incubation with extracellular matrix

Trypomastigotes (1x10^9^/mL) were incubated with 10 mg/mL ECM (Gibco) for 2 h, unless otherwise stated, at 37°C and 5% CO_2_. After incubation, parasites were washed twice in PBS containing 5 mM NaF, 2 mM Na_3_VO_4_, 50 μM Na β-glicerophosphate, 1 mM PMSF and protease inhibitor cocktail (Sigma-Aldrich), and kept at -80°C until used.

### Nitric Oxide quantification

After incubation with ECM, parasites were centrifuged (10,000 x G, 5 minutes) and the supernatant separated for nitric oxide quantification, as described by the manufacturer (Measure-iT High-Sensitivity Nitrite Assay Kit, Invitrogen)

### NOS activity assay

Nitric oxide synthase activity was determined by following the conversion of L[^3^H]-arginine to L[^3^H]-citrulline in *T*. *cruzi* cell lysates (5x10^8^), accordingly to the manufacturer (Cayman), and as previously described [34]. The presence of [^3^H]-citrulline was confirmed by thin layer chromatography and radioactivity measurement of the spots, as described [34].

### Determination of cGMP levels

cGMP was measured in *T*. *cruzi* extracts (5x10^8^ cells) accordingly to the commercial EIA test Biotrak instructions (GE Healthcare). Due to the intrinsic presence of extracellular matrix proteins in some of the experimental assays specific activity could not be calculated and, thus, results are expressed in total femtomoles produced.

### Total S-NO quantification

Total S-NO was quantified by the Saville-Griess method, as described elsewhere [53]. Briefly, the parasite pellet (10^9^) was lysed in 20 mM Tris-HCl buffer, pH 7.4, containing 0.1% Triton X-100, 5 mM NaF, 2 mM Na_3_VO_4_, 50 μM Na β-Glycerophosphate, 1 mM PMSF and protease inhibitor cocktail (Sigma-Aldrich) and centrifuged (10 minutes at 14,000 x G, 4°C). Twenty μL of supernatant were then added to 180 μL reaction buffer (57 mM Sulfanilamide, 1.2 mM N-(1-Naphthyl) ethylenediamine dihydrochloride in PBS, pH 7.4) and the reaction started by the addition of 100 μM HgCl_2_. After 30 minutes at room temperature and in the dark, the absorbance at 496 nm was measured. Controls without HgCl_2_ were included to account for NO already present in the sample. The amount of total S-NO was estimated against a standard curve with S-nitrosoglutathione.

### Determination of L-Arginine and L-Citrulline contents

The parasite pellet was lysed in H_2_O: methanol (1:1, v:v), centrifuged for 10 minutes at 14,000 x G, 4°C and the supernatant collected was dried in SpeedVac. The resulting dried pellet was resuspended with 200 μL MilliQ water and centrifuged (10 minutes at 14,000 x G, 4°C) to remove impurities. The supernatant was analyzed by a capillary electrophoresis system (model PA 800, Beckman Coulter Instruments, Fullerton, USA), equipped with DAD detector and a temperature control device. Data acquisition and treatment were carried out by the vendor software (32 Karat Software version 8.0, Beckman Coulter). A fused silica capillary (Polymicro Technologies, Phoenix, USA) of 50.2 cm total length, 40.0 cm effective length and 50 μm i.d. was used. The capillary was preconditioned as follows: 1 mol.L^-1^ NaOH (5 minutes/20 psi), MilliQ water (5 min/20 psi) and background electrolyte (BGE) (5 minutes/20 psi). The BGE was comprised of 50 mmol.L^-1^ of sodium dihydrogenophosphate at pH 2.5, adjusted with phosphoric acid. Samples were injected hydrodynamically by applying a 0.5 psi pressure during 2 s. The conditions applied during separation were voltage of 25 kV and detection at 200 nm. To quantify arginine and citrulline in the samples, analytical curves were constructed with background electrolyte the linear range of 50–200 mg.mL^-1^ and 1–50 mg.mL^-1^, respectively. Imidazole was used as internal standard (50 mg.mL^-1^).

### Immunofluorescence

After incubation with ECM and subsequent washes, parasites were fixed in 2% paraformaldehyde for 15 minutes at room temperature, pelleted by centrifugation (5,000 x G for 5 minutes), resuspended in PBS and added to a cover glass and left to dry for 16 hours at room temperature. After permeabilization of the parasites (PBS containing 1% BSA and 0.1% Triton X-100 for one hour at 37°C), anti-nitrosocysteine (rabbit, 1:200), anti-nitro-tyrosine (rabbit, 1:500) or anti-alpha-tubulin (mouse, 1:500) were added and incubated for 2 h at 37°C. After exhaustive washes with PBS containing 1% BSA, the correspondent secondary antibodies were added (anti-rabbit Alexa 555 conjugated, 1:500; anti-mouse FITC conjugated, 1:100), followed by one hour incubation at 37°C. After successive washes in PBS-1% BSA, the slides were mounted in a solution containing 50% glycerol, 50% milliQ H_2_O and 10 μg DAPI. The images were taken on an ExiBlue camera (Qimaging) coupled to a Nikon Eclipse E 600 optical microscope and deconvoluted using the software Huygens Essential (Scientific Volume Imaging).

### Immunoblotting

Proteins from the parasite were extracted with Laemmli Buffer [54] without reducing agents. SDS polyacrylamide gel electrophoresis was performed in a 6–16% gradient polyacrylamide gel and transferred to a 0.45 μm nitrocellulose membrane for 16 hours at 15 V. The membrane was blocked in 5% BSA and incubated with the primary antibody (anti-nitrosocysteine or anti-nitro-tyrosine, produced in rabbit, 1:2000), washed thrice in PBS-0.1% Tween 20, incubated with secondary antibody (anti-rabbit conjugated with HRP, 1:8000 dilution), washed five times in PBS-0.1% Tween 20 and developed by electrochemiluminescence.

### Biotin switch

Conversion of protein SNO to biotinylated groups was performed as described [20]. Briefly, parasites were lysed by sonication in 25 mM HEPES, 50 mM NaCl, 0.1 mM EDTA, 1% NP-40, 5 mM NaF, 2 mM Na_3_VO_4_, 50 μM Na β-glicerophosphate, 1 mM PMSF and protease inhibitor cocktail (Sigma), then clarified by centrifugation at 14,000 x G for 10 min at 4°C. The supernatant was blocked in 250 mM Hepes-NaOH buffer, pH 7.7, containing 1 mM EDTA, 0.1 mM Neocuproine, 2.5% SDS and 20 mM MMTS for 20 minutes at 50°C, protected from light. Proteins were then precipitated by the addition of six volumes of ice cold acetone for one hour at ^-^20°C. After successive washes in 70% acetone, the precipitate was resuspended in 250 mM Hepes-NaOH buffer, pH 7.7, containing 1 mM EDTA, 0.1 mM neocuproine, 1% SDS, 4 mM Biotin-HPDP and 1 mM sodium ascorbate. The mix was incubated for one hour at 25°C at dark. Proteins were then precipitated in ice cold acetone for one hour at -20°C and washed extensively in 70% acetone.

Biotin-containing proteins were prepared for sequencing as described [21] Proteins were digested by trypsin (sequencing grade 1:100, mass/mass) for 16 hours at 37°C and the reaction stopped by the addition of 0.5 mM PMSF. Then, biotin-containing proteins were pulled-down using streptavidin beads. After 2 hours incubation at room temperature under gentle shaking, the beads were washed thrice in 20mM Tris-HCl buffer pH 7.7, containing 1 mM EDTA and 0.4% Triton X-100. Proteins were eluted with 5 mM ammonium bicarbonate containing 150 μL of 50 mM 2-mercaptoethanol for 5 minutes. Sequencing was determined (Veritas Life Sciences, Brasil) using a LTQ-Orbitrap coupled to nLC-MS/MS. Acquired data were automatically processed by CPAS (Computational Proteomics Analysis System) [55] and only peptides with high quality were considered (expected score <0.2). The TryTripDB was used for protein search combining Esmeraldo-like, non-Esmeraldo-like and unassigned.

For Mucin II validation, proteins were biotinylated as described above, ressuspended in Tris-HCl 20 mM and incubated at 12°C for 16 hours with streptavidin beads. After three successive washes, the beads were ressuspended in Laemmli buffer without reducing agents, incubated at 100°C for 5 minutes, followed by SDS-PAGE using a 6–16% gradient gel. The proteins were transferred to a nitrocellulose membrane and the immunoblotting was performed using anti-rabbit Mucin II antibodies (1: 500 in PBS- 5% BSA).

### Immunoprecipitation and identification of tyrosine-nitrated proteins

Parasites were lysed by sonication using 100 μL RIPA buffer, containing 5 mM NaF, 2 mM Na_3_VO_4_, 50 μM Na β-Glicerophosphate, 1 mM PMSF and protease inhibitor cocktail (Sigma). Samples were diluted in 1.4 mL of 20 mM Tris-HCl buffer, pH 7.4, containing the same concentration of inhibitors. After centrifugation at 14,000 x G for 10 minutes at 4°C, the supernatant was added to protein A- Agarose with the desired antibody or normal serum (control) or to covalent-linked antibody-Agarose (anti-nitro-tyrosine-resin). After 16 h at 12°C, the beads were washed with 20 mM Tris-HCl buffer, pH 7.4 containing 0.1% Triton X-100 and the bound material eluted with Laemmli buffer without reducing agent. In the particular case of immunoprecipitation using covalent-linked anti-nitro-tyrosine antibodies, the elution was performed by incubation in 5 M acetic acid for 5 minutes. The eluted proteins were identified by a commercial facility (Veritas Life Sciences, Brasil), as described above.

## Results

It was previously shown that *T*. *cruzi* is able to produce •NO via a putative tcNOS [34] although no NOS ortholog can be found on the parasite genome. The synthesis of •NO was then first confirmed in *T*. *cruzi* extracts of the non infective (epimastigote) and infective (trypomastigote) stages by measuring the conversion of ^3^H-L-arginine to ^3^H-L-citrulline by thin layer chromatography, as described [34] and the product of the reaction confirmed by capillary electrophoresis. Importantly, approximately 260 fold-enrichment in NOS activity was obtained after partial purification of the enzyme from epimastigotes, as described [34].

**Fig 1:**
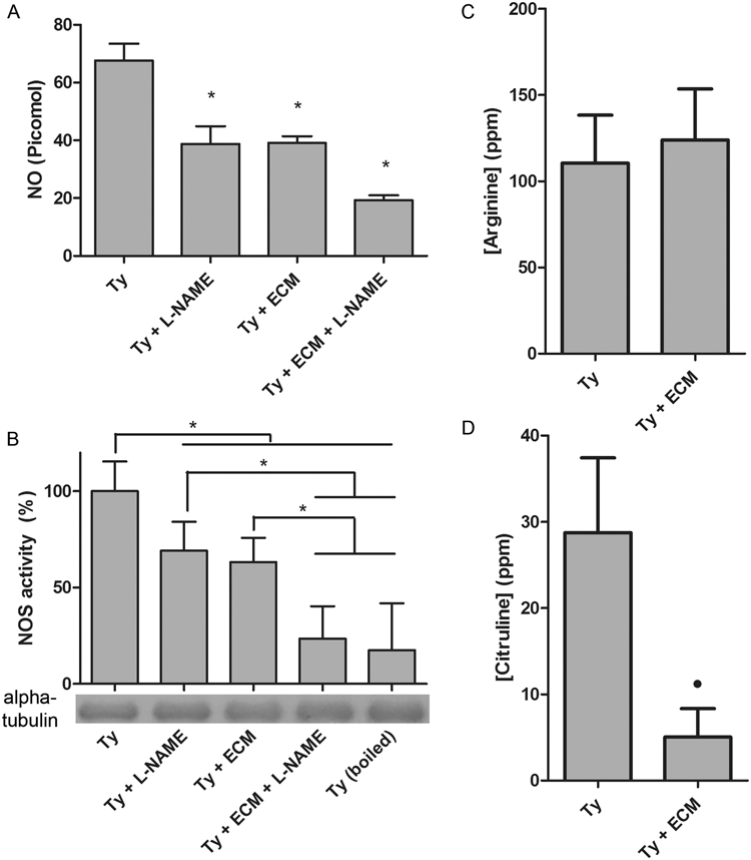
Nitric oxide synthase activity during *Trypanosoma cruzi* trypomastigotes adhesion to extracellular matrix. Trypomastigotes (1x10^9^) were incubated with ECM (1.5 mg) in phenol red free-MEM, supplemented with 2% FBS, for 2 h at 37°C and 5% CO_2._ The inhibitor L-NAME (10 mM) was added after the extract preparation. (A) •NO concentration in the medium was measured employing a modified fluorescent reaction of DAF-2 (Measure-iT High-Sensitivity Nitrite Assay Kit). (B) NOS activity was measured in *T*. *cruzi* extracts by the conversion of [^3^H]arginine to [^3^H]citrulline in *T*. *cruzi* extracts. The immunoblots were probed with anti-tubulin antibody to guarantee that the protein loading in each track was similar. The levels of intracellular L-Arginine (C) and L-citrulline (D) were obtained by the capillary electrophoresis method. Points marked with an asterisk represent a p<0.001 (one-way ANOVA) and with a dot represent p<0.001 in unpaired t-Student test. Results in (A) and (B) are the mean of three independent experiments and (C) and (D) are the mean of five independent experiments.

Even though the identity of the enzyme remains elusive, the possible involvement of •NO signaling during *T*. *cruzi* binding to ECM was pursued. Trypomastigotes were incubated with purified ECM for to 2 h and the amount of extracellular •NO was quantified (Fig. 1A). The extracellular •NO concentration dropped 43% under this condition, in the same order of magnitude observed when trypomastigote extracts were incubated with 10 mM L-NAME, a NOS inhibitor (Fig. 1A). The simultaneous incubation with ECM and L-NAME leads to an even higher inhibition of •NO production (approximately 72%). The data strongly suggest that interaction of the parasites with ECM hinders •NO responses.

Accordingly, 37% decrease in NOS activity was observed in parasite extracts previously incubated with ECM, as compared to parasites incubated in the absence of ECM under the same experimental conditions (Fig. 1B). Partial or total inhibition of •NO production by 10 mM L-NAME or by boiling the cellular extracts for 10 minutes at 100°C, respectively, confirmed that the •NO measured is a product of an enzymatic activity (Fig. 1B). The enzymatic activity was reduced to 9% by the addition of 50 mM L-NAME. Furthermore, changes in L-arginine/L-citrulline ratio upon incubation with ECM strengthen the evidence of declining NOS activity upon parasite adhesion to ECM (Fig. 1C, D). Whereas Intracellular concentration of L-citrulline decreased 83% upon adhesion of trypomastigotes to ECM, no significant change was observed in the L-arginine levels. This could be attributed to the contribution of other metabolic routes, but it is important to note that *T*. *cruzi* lacks a pathway to convert citrulline to arginine (i.e. arginase, an enzyme of the urea cycle, is absent) [56], which strongly suggests that the decline in the L-citrulline levels might be, at least in part, a consequence of NOS activity inhibition.

**Fig 2:**
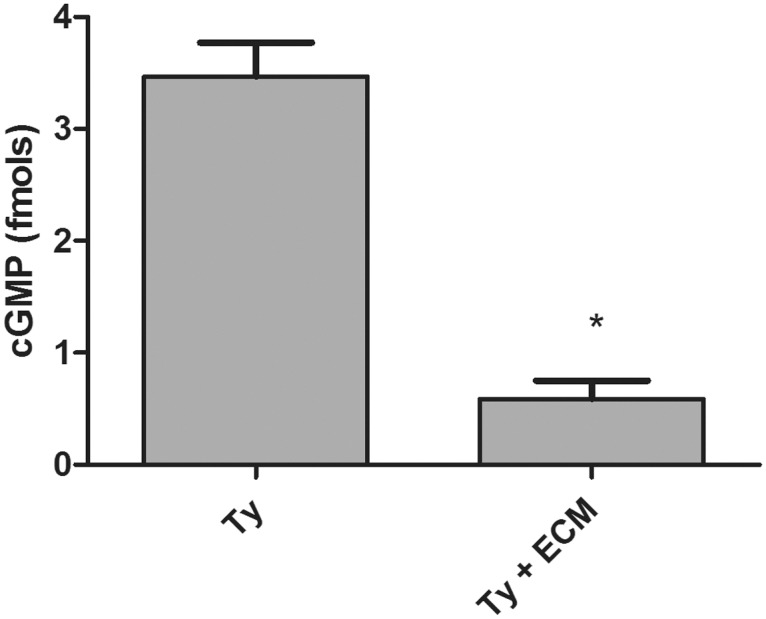
cGMP responses during *Trypanosoma cruzi* trypomastigotes adhesion to extracellular matrix. Trypomastigotes (1x10^9^) were incubated with ECM (1.5 mg) in phenol red free-MEM, supplemented with 2% FBS, for 2 h at 37°C and 5% CO_2_. cGMP levels were measured in *T*. *cruzi* extracts by competitive ELISA. The asterisk represents a p<0.001 according to *t-Student* test. Values are the mean of four independent experiments.

Since biological signaling by •NO is primarily mediated by activation of guanylyl cyclase, the production of cGMP in trypomastigotes incubated or not with ECM was quantified (Fig. 2). The levels of cGMP production fell from 3.5 to 0.6 fmoles after adhesion of the parasite to ECM (Fig. 2). Taken together, the findings strongly suggest that parasite adhesion to ECM leads to inhibition in •NO production, consequently deactivating a classical •NO signaling pathway.

**Fig 3:**
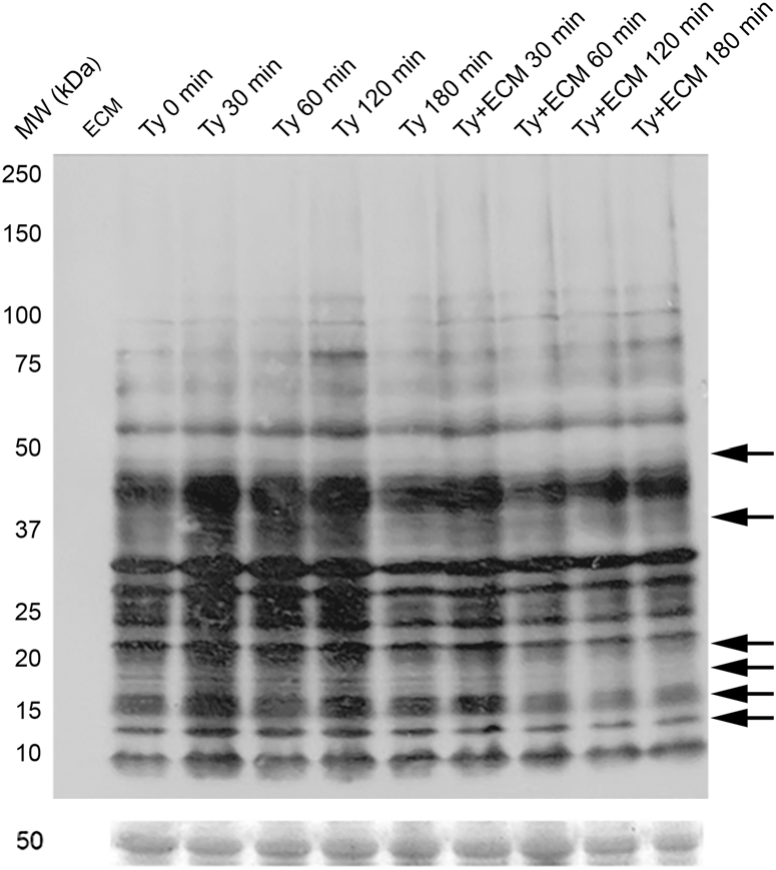
S-nitrosylation pattern during *Trypanosoma cruzi* trypomastigotes adhesion to extracellular matrix. Trypomastigotes (1x10^9^) were incubated with ECM (1.5 mg) in phenol red free-MEM, supplemented with 2% FBS, at 37°C and 5% CO_2_, for different incubation times. Proteins (50 μg) were resolved in 6–16% gradient SDS-PAGE. The immunoblotting was performed using the antibodies: rabbit anti-SNO 1:2,000, and the secondary HRP conjugated anti-rabbit 1:8,000. The Ponceau’s staining was used as a load control. Arrows indicate protein bands in which the signal intensity is decreased on the group incubated with the extracellular matrix in relation to the equivalent group incubated with growth media alone. The figure is representative of three experiments.

To check whether parasite adhesion to ECM would modulate protein S-nitrosylation (SNO) and tyrosine nitration, immunological assays were performed employing anti-S-nitroso-cysteine and anti-3-nitro-tyrosine antibodies. Immunoblotting experiments reveal a time-dependent decrease of SNO in specific bands, mainly in the 37 kDa region, but also noticeable in protein bands at the 47, 20, 18, 15 and 13 kDa regions (Fig. 3).

**Fig 4:**
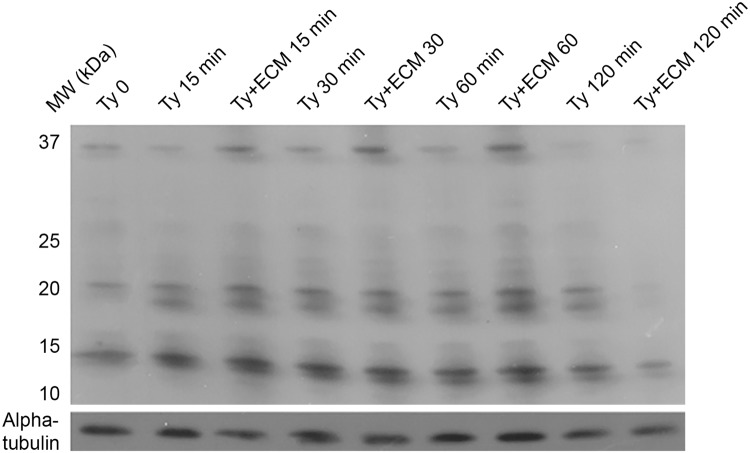
Tyrosine nitration pattern during *Trypanosoma cruzi* trypomastigotes adhesion to extracellular matrix. Trypomastigotes (1x10^9^) were incubated with ECM (1.5 mg) in phenol red free-MEM, supplemented with 2% FBS, at 37°C and 5% CO_2_, for different incubation times. Proteins (50 μg) were resolved in 6–16% gradient SDS-PAGE. The immunoblotting was performed using the antibodies: rabbit anti-TyrNO_2_ 1:2,000, mouse anti-α-tubulin 1:5,000 as a load control and the secondary HRP conjugated anti-rabbit or anti-mouse 1:8,000 antibodies. The figure is representative of three independent experiments.

Differently from SNO, the number of nitrated-proteins detected by anti-3-nitro-tyrosine was considerably less and differences in tyrosine-nitrated proteins were not significant at the first hour of the experiment (Fig. 4). However, the levels of tyrosine-nitrated proteins were extensively reduced at 2 h incubation, affecting proteins in the range of 10 to 37 kDa (Fig. 4).

**Fig 5:**
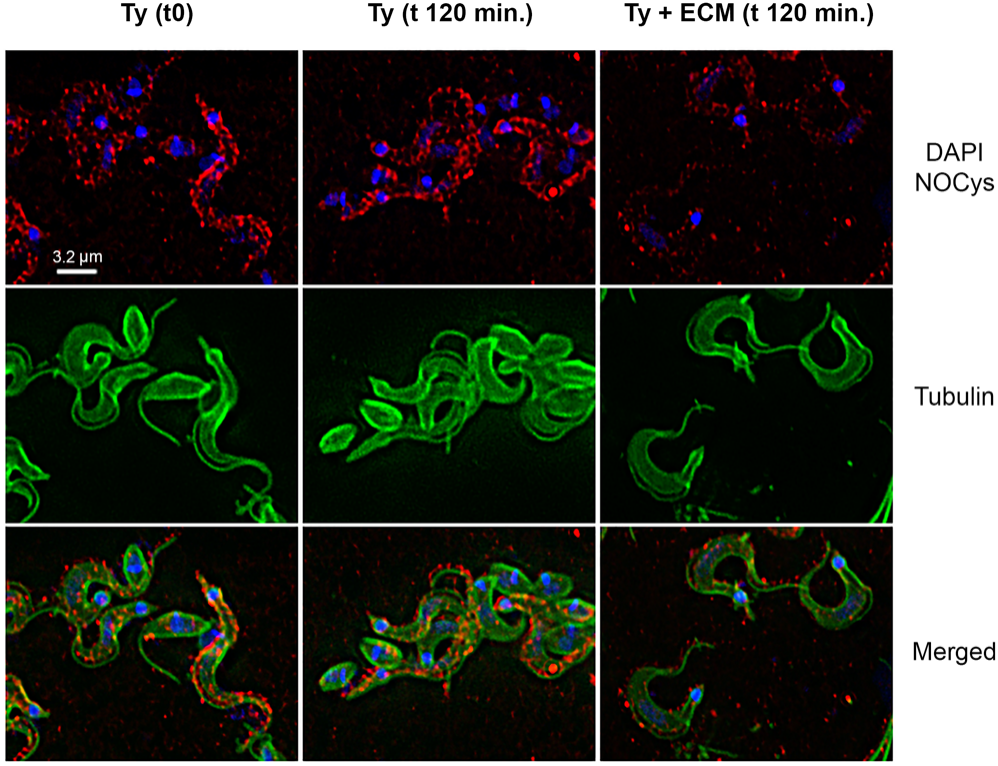
Presence of S-nitrosylated proteins in *Trypanosoma cruzi* trypomastigotes during adhesion to the extracellular matrix. Trypomastigotes (1x10^9^) were incubated with ECM (1.5 mg) in phenol red free-MEM, supplemented with 2% FBS, for 2 h at 37°C and 5% CO_2_. Parasites were submitted to the immunofluorescence protocol. S-nitrosylated proteins are stained in red, alpha-tubulin in green, and nucleus and kinetoplast in blue (DAPI). The white bar represents 3.2 μm.

**Fig 6:**
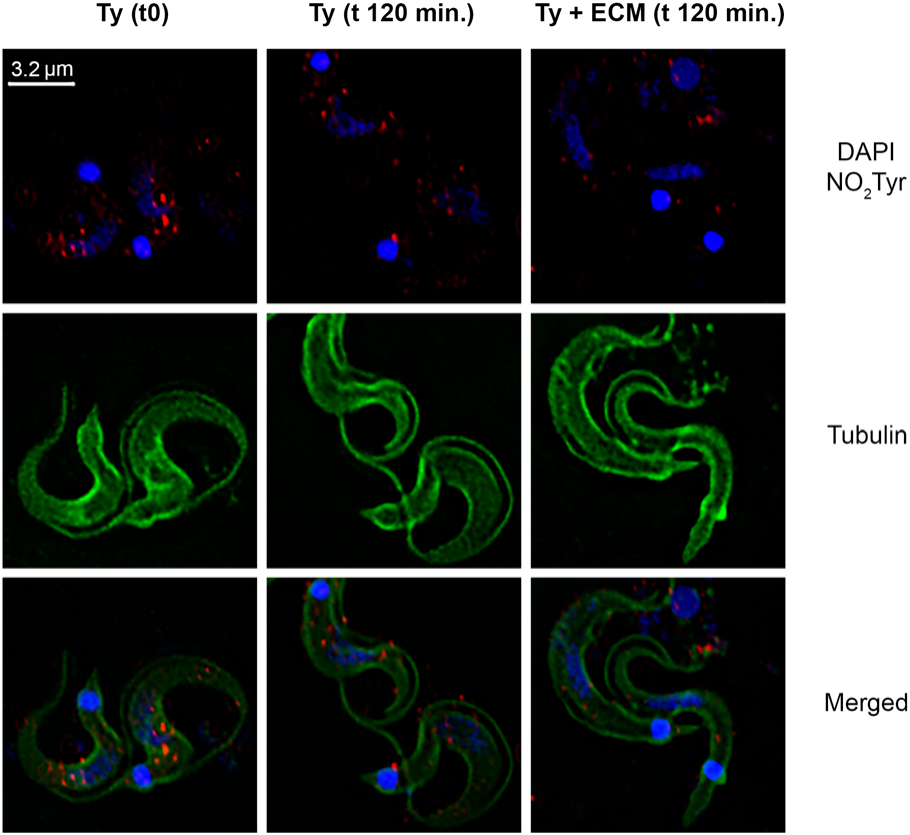
Presence of tyrosine nitrated proteins in *Trypanosoma cruzi* trypomastigotes during adhesion to the extracellular matrix. Trypomastigotes (1x10^9^) were incubated with ECM (1.5 mg) in phenol red free-MEM, supplemented with 2% FBS, for 2 h at 37°C and 5% CO_2_. Parasites were submitted to the immunofluorescence protocol. Tyrosine nitrated proteins are stained in red, alpha-tubulin is stained in green, nucleus and kinetoplast are stained in blue (DAPI). The white bar represents 3.2 μm. The image is representative of two experiments.

Likewise, the general decrease in SNO and nitrated-proteins can be observed by immunofluorescence microscopy. Paraformaldehyde-fixed *T*. *cruzi* trypomastigotes previously incubated with ECM for 2 h showed a significant decrease in the immune reaction for both S-nitrosylation (Fig. 5) and tyrosine nitration of proteins (Fig. 6). Indeed, image pixels/spots quantification showed a reduction higher than 50% and around 30% in the immunoreaction for S-nitrosylated and for tyrosine nitration proteins, respectively, when parasites were incubated with ECM.

**Fig 7:**
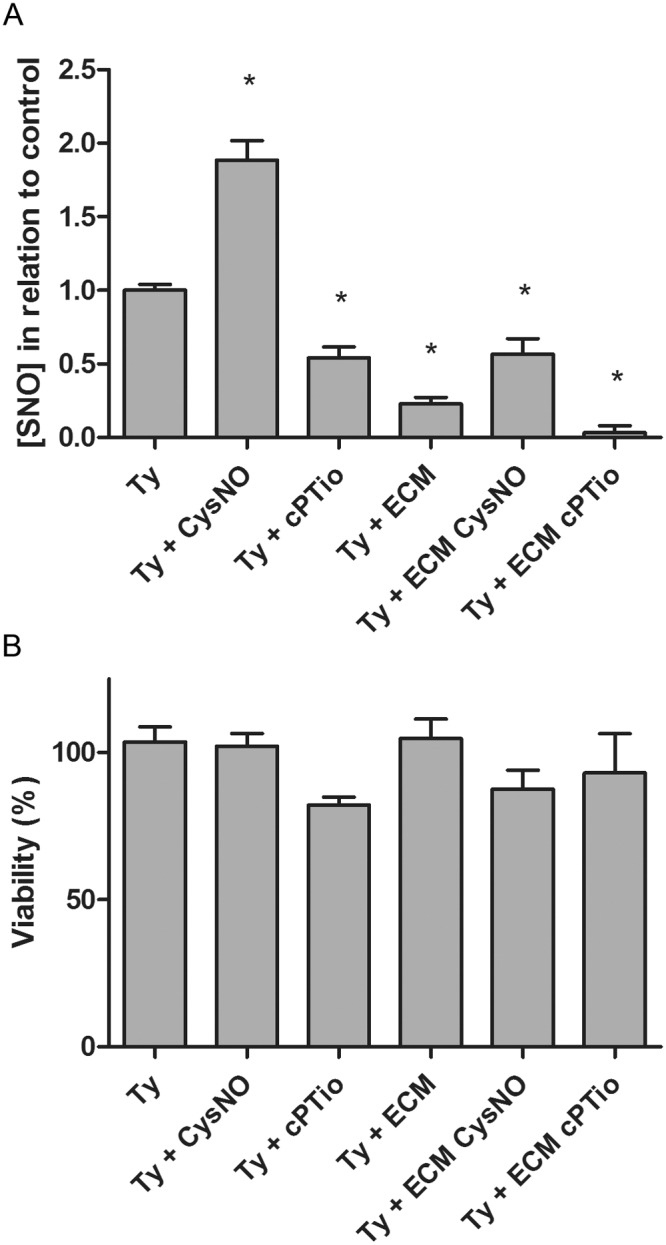
The effect of nitric oxide availability on *Trypanosoma cruzi* protein S-nitrosylation. Trypomastigotes (1x10^9^) were incubated with ECM (1.5 mg) in phenol red free-MEM, supplemented with 2% FBS, for 2 h at 37°C and 5% CO_2_, in presence or absence of 100 μM cPTIO or CysNO. (A) Total protein S-NO was quantified in the parasite lysate using the Saville-Griess method. Asterisks represent a p<0.01 according to one-way ANOVA. (B) Parasite cell viability (1x10^7^/well in a 96 well plate) was carried out by following the reduction of WST-1 reagent (Roche) as described by the manufacturer. Differences are not statistically significant. Values are the mean of three independent experiments.

In order to confirm the adhesion effect on protein S-nitrosylation, total S-nitrosylated proteins in *T*. *cruzi* extracts were quantified by the Saville-Griess method [53]. A pronounced decrease of 87% was observed in the total SNO trypomastigote proteins when parasites were incubated with ECM, as compared to the control (Fig. 7A). As additional controls, parasites were also incubated in the presence or absence of 100 μM CysNO (•NO donor) or 100 μM cPTio (•NO scavenger). As expected, increasing •NO availability led to an improvement in SNO, as well removing •NO from the system resulted in SNO decrease (Fig. 7A). Also, addition of CYsNO to parasites incubated with ECM did not restore the levels observed for trypomastigotes incubated with CYsNO only, showing the predominance of the ECM effect. On the other hand, cPTio added to ECM-treated parasites reduced even more the amount of S-nytrosylated proteins. The different treatments did not affect parasite viability (Fig. 7B).

Preliminary data using the biotin-switch technique and mass spectrometry further confirmed the presence of S-nitrosylated proteins in *T*. *cruzi*. Although a low number of SNO proteins were detected, an even lower amount was present in ECM-incubated trypomastigotes, as predicted by the experiments herein described.

Examples of putative modified proteins that have been identified under different conditions were: (1) calpain-like cysteine peptidase, retrotransposon hot spot protein, surface protease GP63, trans-sialidase and mucin TcMUCII, in both untreated and ECM-incubated parasites; (2) fucose kinase, glycerophosphate mutase and kinesin K39 only in ECM-incubated parasites; (3) DGF-1, fatty acid elongase and helicase only in untreated parasites. Additionally, 27 hypothetical S-nitrosylated proteins were detected in ECM-treated (7) or untreated (20) trypomastigotes. However, it must be emphasized that the presence or absence of a modification in a particular protein due to the incubation of the parasite with ECM needs validation in each case, due to the possibility of a nonspecific binding during the enrichment of the SNO proteins.

**Fig 8:**
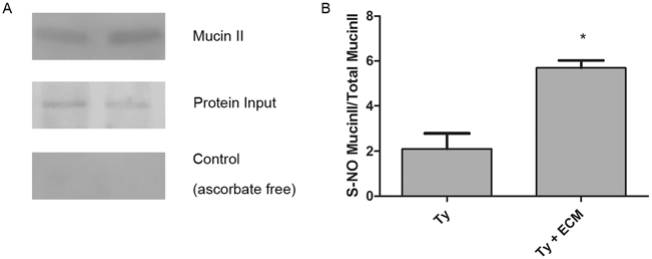
Validation of *T*. *cruzi* cysteine nitrosylated proteins. (A). S-Nitrosylated proteins from ECM-incubated and ECM-non-incubated parasites were isolated by biotin switch and streptavidin pull down methodology and submitted to immunoblotting using anti-mucin II antibodies. Control of protein input and negative control (the biotin switch methodology in the absence of ascorbate) are shown. *T*. *cruzi* control (Ty) and treated with ECM for 2 hours (Ty + ECM). (B) Quantification of the results presented in (A). The asterisk represents a p<0.001 according to *t-Student* test.

The existence of S-nitrosylation in *T*. *cruzi* proteins was validated for mucin TcMUCII. SNO proteins were converted to biotin-containing proteins, pulled-down by streptavidin beads as described in Materials and Methods, and the biotinylated-proteins were subjected to immunoblotting using anti-rabbit Mucin II antibodies (Fig. 8A). As a negative control, proteins prepared by the biotin switch method in the absence of ascorbate gave no reactivity with anti-mucin II antibodies (Fig. 8A). A significant increase in the level of S-nitrosylation was detected in ECM-treated trypomastigotes in relation to the untreated parasites (Fig. 8B) and taking into account the protein loaded in each case.

In relation to the tyrosine-nitrated modifications, immunoprecipitated proteins with anti-nitro-tyrosine antibodies were identified by nLC-MS/MS. A number of putative nitrated targets identified decreased in ECM-incubated trypomastigotes (Table 1). Hypothetical and ribosomal proteins comprise the majority of the sequences obtained in untreated trypomastigotes. Also, the majority of the identified proteins were detected in ECM- treated or untreated parasites.

**Table 1:** Putative Tyrosine nitrated *T*. *cruzi* proteins identified by nLC/MS-MS after sample enrichment by immunoprecipitation.

Group	Protein	Gene ID	Function	Theoretical	
				MM (Da)	pI
Ty	10 kDa heat shock protein, putative	TcCLB.508209.90	Protein folding	10701	9.07
Ty	40S ribosomal protein S10, putative	TcCLB.506679.140	Translation	18250	10.8
Ty	40S ribosomal protein S14, putative	TcCLB.508823.50	Translation	15568	10.6
Ty	40S ribosomal protein S24E, putative	TcCLB.507681.150	Translation	15668	11.7
Ty	40S ribosomal protein S3a-1 OS	TcCLB.510999.39	Translation	28288	11
Ty	40S ribosomal protein S4, putative	TcCLB.509683.117	Translation	30876	11.1
Ty	40S ribosomal protein S6, putative	TcCLB.507709.50	Translation	21400	11.4
Ty	60S ribosomal protein L10a, putative	TcCLB.506963.10	Translation	25146	10.2
Ty	60S ribosomal protein L13, putative	TcCLB.508153.280	Translation	25294	11.9
Ty	60S ribosomal protein L18, putative	TcCLB.506181.50	Translation	34028	11.1
Ty	60S ribosomal protein L23a, putative	TcCLB.508175.146	Translation	21268	11.5
Ty	60S ribosomal protein L28, putative	TcCLB.510101.30	Translation	16368	12.2
Ty	60S ribosomal protein L6, putative	TcCLB.507709.50	Translation	21400	11.4
Ty	60S ribosomal protein L7a, putative	TcCLB.510835.40	Translation	34550	11.8
Ty, Ty+ECM	ADP, ATP Carrier protein 1, mitocondrial precursos, putative	TcCLB.506211.160	Transport	34955	10.3
Ty, Ty+ECM	alpha tubulin, putative	TcCLB.411235.9	Cytoskeleton	49800	4.7
Ty	ATP synthase, epsilon chain, putative	TcCLB.506945.240	Metabolism	20246	6.08
Ty, Ty+ECM	beta tubulin, putative	TcCLB.506563.40	Cytoskeleton	49701	4.43
Ty	beta-fructofuranosidase-like protein	TcCLB.506705.70	Metabolism	53584	7.79
Ty	chaperonin HSP60, mitochondrial precursor	TcCLB.510187.551	Protein folding	30553	9.15
Ty, Ty+ECM	cytochrome c, putative	TcCLB.506949.50	Metabolism	12234	9.88
Ty, Ty+ECM	elongation factor 1-alpha, putative	TcCLB.511369.30	Translation	30736	9.33
Ty	enoyl-CoA hydratase, mitochondrial precursor, putative	TcCLB.508185.10	Metabolism	28818	8.91
Ty, Ty+ECM	glyceraldehyde 3-phosphate dehydrogenase, putative	TcCLB.506943.50	Metabolism	39033	9.2
Ty, Ty+ECM	glycosomal malate dehydrogenase, putative	TcCLB.506503.69	Metabolism	34068	8.88
Ty	guanosine monophosphate reductase, putative	TcCLB.506519.130	Metabolism	52311	9.05
Ty, Ty+ECM	heat shock 70 kDa protein, putative	TcCLB.511211.170	Protein folding	73299	5.22
Ty, Ty+ECM	histone H2A, putative	TcCLB.510525.80	DNA packaging	14354	11.9
Ty, Ty+ECM	histone H2B, putative	TcCLB.511635.10	DNA packaging	12361	12.2
Ty, Ty+ECM	histone H3, putative	TcCLB.505931.50	DNA packaging	14762	11.9
Ty, Ty+ECM	histone H4, putative	TcCLB.510351.20	DNA packaging	11170	11.7
Ty+ECM	hypothetical protein, conserved (pseudogene)	TcCLB.511821.179	N/A	143829	4.19
Ty	hypothetical protein, conserved	TcCLB.504089.70	N/A	12959	8.48
Ty	hypothetical protein, conserved	TcCLB.504001.10	N/A	13465	9.78
Ty+ECM	hypothetical protein, conserved	TcCLB.510877.40	N/A	24454	10.2
Ty	hypothetical protein, conserved	TcCLB.510143.5	N/A	7311	6.52
Ty	hypothetical protein, conserved	TcCLB.508719.70	N/A	44107	10.6
Ty, Ty+ECM	kinetoplast DNA-associated protein, putative	TcCLB.508719.60	DNA packaging	14361	12.1
Ty	kinetoplast-associated protein 3	TcCLB.509791.120	DNA packaging	21245	11.9
Ty	mitochondrial oligo_U binding protein TBRGG1, putative	TcCLB.507927.20	RNA processing	97266	8.8
Ty	poly(A)-binding protein, putative	TcCLB.508461.140	RNA processing	61411	9.69
Ty	ribosomal protein l35a, putative	TcCLB.506559.470	Translation	16892	12.1
Ty	ribosomal protein L36, putative	TcCLB.509671.64	Translation	12948	12.1
Ty, Ty+ECM	ribosomal protein S25, putative	TcCLB.504105.94	Translation	12351	11.2
Ty	ribosomal protein S7, putative	TcCLB.506593.19	Translation	23915	11.9
Ty	RNA-binding protein, putative	TcCLB.508413.50	RNA Processing	31097	9.78

Tyrosine nitrated *T*. *cruzi* proteins after 2 h incubation of the parasite in the presence of bovine serum albumin (Ty) or extracellular matrix (Ty+ECM).

**Fig 9:**
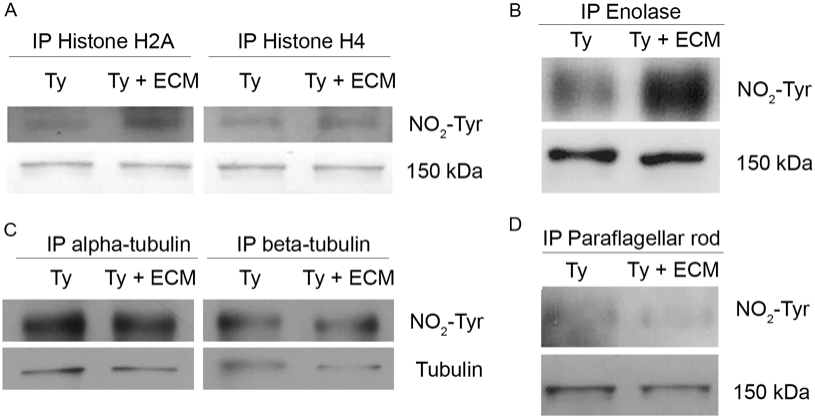
Validation of *T*. *cruzi* tyrosine nitrated proteins. Proteins of interest were immunoprecipitated (IP) with specific antibodies (anti histone H2A, anti-histone H4, anti-enolase, anti-alpha tubulin, anti-beta tubulin or anti-paraflagellar rod proteins antibodies) and submitted to immunoblotting employing anti-nitro-tyrosine antibodies. A 150 kDa band of the blotting (A, B and D) or anti-tubulin antibodies (C) are shown as controls of the protein loading. *T*. *cruzi* control (Ty) and treated with ECM for 2 hours (Ty + ECM).

To confirm this post-translational modification, histone 2A, histone 4B, enolase, alpha-tubulin, beta-tubulin and paraflagellar rod proteins (PAR) were selected. Modified histones and tubulins were detected herein (Table 1), enolase was included since nitrated-enolase was already described in the literature [57] and PAR was chosen as a negative control of the method. The mentioned proteins were immunoprecipitated with commercial specific antibodies (except for anti-PAR monoclonal antibody prepared in the laboratory [50]) followed by Western blot developed with anti-nitro-tyrosine antibodies. An increase in the nitrosylation levels of enolase and histones 2A and 4 were observed after the incubation with ECM (Fig. 9A, B). No changes in the nitration levels were observed when a similar experiment was performed using anti-alpha and beta-tubulin antibodies, while no reactivity was detected with paraflagellar proteins immunoprecipitated with anti-PAR monoclonal antibody (Fig. 9C, D). The results have shown that in spite of the general down regulation of protein S-nitrosylation and nitration upon incubation of the parasites with ECM, there is a specific response for each protein, including the stimulation of the nitration levels of enolase and histones 2A and 4B.

Taken together, the results herein presented strongly suggest that *T*. *cruzi* responds to the interaction with ECM by the involvement of •NO regulated pathways.

## Discussion

Nitric oxide is a key signaling molecule affecting many biological activities. Human parasites such as *T*. *cruzi* are exposed to the anti-parasitic •NO produced by the host but also to its own •NO. Since the interaction of *T*. *cruzi* trypomastigotes with ECM is an essential step during the infective process [2,48,49], the *in vitro* model in the absence of host cells has been used to study the role of •NO in the parasite response to the interaction. ECM is a very dynamic structure and its relevance in •NO signaling was shown, for example, by thrombospondin-1 inhibition [58] of the •NO pathway in vascular cells. Additionally, the relevance of ECM to *T*. *cruzi* signaling was previously shown by changes in parasite protein phosphorylation levels [50].

Endogenous nitric oxide is predominantly produced in *T*.*cruzi* by enzyme catalysis, probably by NOS, as described [34] since the addition of the inhibitor L-NAME drastically reduces •NO production (Fig. 1A, B). In addition to •NO, the reaction produces L-citrulline from the substrate L-arginine. Of note, a strong reduction of citrulline, but not of arginine, was measurable in ECM-incubated trypomastigotes, suggestive of an inhibited NOS activity, although other possibilities such as its utilization in another activated metabolic route could not be ruled out. Arginine, on the other hand, is a substrate for protein synthesis and a precursor of •NO and other important metabolites as phosphoarginine, an energy buffer synthesized in *T*. *cruzi* by arginine kinase. Due to its relevance, it was previously suggested that arginine concentration under different external conditions may be buffered by TcAAP3, a specific permease [59].

Adhesion of trypomastigotes to ECM resulted in a remarkable inhibition not only on NOS activity but also in •NO and cGMP concentrations (Fig. 1, 2). However, a direct correlation between •NO levels and cGMP concentrations is difficult to make since the amount of •NO that may activate cGMP synthesis in trypomastigotes is unknown, due to the fact that no typical guanylyl cyclase is present in the *T*. *cruzi* genome. Moreover, the putative cGMP synthetic activity of an ubiquitous adenylyl cyclase remains non characterized [37]. Additionally, cGMP concentrations would depend on its degradation by a soluble dual-specificity phosphodiesterase (TcrPDEC) [38,39,40,41]. Presumably, the downstream signal transmission may be dependent on a protein kinase A activated by cGMP, as described for *T*. *brucei* [45] and *Leishmania* [46].

The decrease in total nitration and S-nitrosylation levels of proteins as described here for ECM-incubated trypomastigotes probably reflects the lower level of nitrosative stress. Changes in S-nitrosylated proteins were easily noticed by Western blot experiments after 30 minutes incubation of the parasites with ECM, with a marked decrease after 2 h period time (Fig. 5) and confirmed by immunofluorescence and decrease in the total SNO measured (Figs. 5 and 7). S-nitrosylation of proteins is a key player in diverse biological functions of •NO and is associated with processes such as apoptosis [60] and regulation of numerous signaling pathways, for example, PKC [61] and MAPK [62]. Of interest, S-nitrosylation has been associated with activation and desensitization of the human soluble guanylyl cyclase that possesses 37 cysteine residues (review in [58]). To our knowledge, the only study describing S-nitrosylation in *T*. *cruzi* proteins used •NO-donors to investigate a possible role of the host derived •NO in the inhibition of cruzipain, a cysteine protease important for the parasite infection [33]. However, the relevance of S-nitrosylation in *T*. *cruzi* signaling was not further explored.

Here proteins putatively S-nitrosylated in normal conditions and after interaction of the *T*. *cruzi* with the extracellular matrix were analyzed for the first time, using mucin II to validate the modification (Fig. 8). A number of other interesting targets were identified including some proteins already described as S-nitrosylated with relevant modification in function, such as Dual Specificity Phosphatase (DUSP) [62], Serine-Threonine Protein Kinase [61] and HSP 90 [63]. Interestingly, phosphorylation levels in DUSP and Serine/Threonine Protein Kinase were also modified in ECM-incubated parasites [50], but the possibility that both modifications are somehow related, as happens in other cases, has not been addressed. The results described point out to a possible role of the S-nitrosylation in *T*.*cruzi* signaling pathways and will be further explored.

Tyrosine nitration is not as well understood as S-nitrosylation, although relevant processes seem to be modulated by this covalent modification, such as PKC signaling [30] and protein degradation [28]. The present study was able to identify some of the proteins previously described as potential nitration targets (Table 1), such as enoyl-CoA hydratase [64], glyceraldehyde-3-phosphate dehydrogenase [65], heat shock protein 70 [66] and histone 2A [67]. Furthermore, validation of the data for histone 2A, histone 4, enolase and tubulins was achieved (Fig. 8). In the literature, for example, nitration of histones was associated with the induction of autoimmunity in systemic lupus erythematosus and rheumatoid arthritis [67]. Remarkably, a large number of 40 and 60 S-ribosomal proteins were modified by nitration, but whether this modification affects protein expression in *T*. *cruzi* remains to be elucidated. Nitration of ribosomal proteins was also described in native and differentiated PC12 cells, but tryptophan was identified as the modified amino acid [11].

In summary, the present work was able to confirm previous claims on the existence of enzymatic NOS activity in *T*. *cruzi* and demonstrated that the •NO classic signaling pathway is greatly inhibited in the presence of ECM regarding the synthesis of •NO and cGMP. Furthermore, numerous possible S-nitrosylation and tyrosine nitration targets have been identified, with a total decrease in the level of modified proteins upon interaction of the parasite with ECM. However, in spite of general down regulation of protein S-nitrosylation and nitration, the increase in the S-nitrosylation level of mucin II or in the nitration of enolase or histones 2A and 4, in contrast to the constant nitration levels of alpha and beta-tubulins under any condition, point out to the specificity of the modification for each particular target. The biological relevance of each of these target modifications remains to be explored and may give clues to the function of each target in parasite internalization into host cells. In this regard it must be stressed that •NO availability appears to be essential for parasite motility [68]. Thus, it is tempting to speculate that adhesion of *T*. *cruzi* trypomastigotes to ECM, an obligatory path to reach the host cell, triggering decrease in •NO levels, may also decrease parasite motility, somehow facilitating its binding to the host cell plasma membrane prior to invasion.
